# Cubic Lattice Structures of Ti6Al4V under Compressive Loading: Towards Assessing the Performance for Hard Tissue Implants Alternative

**DOI:** 10.3390/ma14143866

**Published:** 2021-07-10

**Authors:** Sahil Dhiman, Malkeet Singh, Sarabjeet Singh Sidhu, Marjan Bahraminasab, Danil Yurievich Pimenov, Tadeusz Mikolajczyk

**Affiliations:** 1 Department of Mechanical Engineering, Thapar Institute of Engineering and Technology, Patiala 147004, India; 2 Department of Mechanical Engineering, Indian Institute of Technology Ropar, Roopnagar 140001, India; malkeet.singh@iitrpr.ac.in; 3 Surface Engineering for Advanced Materials, Swinburne University of Technology, Hawthorn, VIC 3122, Australia; 4 Department of Mechanical Engineering, Sardar Beant Singh State University, Gurdaspur 14521, India; sarabjeetsidhu@yahoo.com; 5 Nervous System Stem Cells Research Center, Semnan University of Medical Sciences, Semnan 3514799422, Iran; m.bahraminasab@yahoo.com; 6 Department of Tissue Engineering and Applied Cell Sciences, School of Medicine, Semnan University of Medical Sciences, Semnan 3514799422, Iran; 7 Department of Automated Mechanical Engineering, South Ural State University, Lenin Prosp. 76, 454080 Chelyabinsk, Russia; danil_u@rambler.ru; 8 Department of Production Engineering, UTP University of Science and Technology, Al. prof. S. Kaliskiego 7, 85-796 Bydgoszcz, Poland; tami@utp.edu.pl

**Keywords:** porous lattice structures, additive manufacturing, cubic unit cell, compressive strength, finite element analysis, selective laser melting, titanium

## Abstract

Porous Lattice Structure (PLS) scaffolds have shown potential applications in the biomedical domain. These implants’ structural designs can attain compatibility mechanobiologically, thereby avoiding challenges related to the stress shielding effect. Different unit cell structures have been explored with limited work on the fabrication and characterization of titanium-based PLS with cubic unit cell structures. Hence, in the present paper, Ti6Al4V (Ti64) cubic PLS scaffolds were analysed by finite element (FE) analysis and fabricated using selective laser melting (SLM) technique. PLS of the rectangular shape of width 10 mm and height 15 mm (ISO: 13314) with an average pore size of 600–1000 μm and structure porosity percentage of 40–70 were obtained. It has been found that the maximum ultimate compressive strength was found to be 119 MPa of PLS with a pore size of 600 μm and an overall relative density (RD) of 57%. Additionally, the structure’s failure begins from the micro-porosity formed during the fabrication process due to the improper melting along a plane inclined at 45 degree.

## 1. Introduction

Advanced biomaterials are in demand to appropriately repair and substitute human body organs that are damaged or diseased by traumatic or pathologic events [1]. Mechanical properties of the implants made up of such materials ought to be equivalent to that of the adjacent natural bone. Due to the difference in mechanical properties between the implant and natural bone, the stress shielding effect occurs which leads to bone loss and implant loosening [2]. The mechanical properties, therefore, should be tailored to avoid this problem. One way is to induce porosity to the solid implant structure. In the meantime, the porous materials allow biological anchorage with the surrounding bone tissue to adhere and grow through its porous network [3]. Biomaterials can be synthetic or natural and includes polymers, ceramics, and metals. Metallic biomaterials are widely used for hard tissue replacement implants owing to their excellent mechanical properties as compared to their non-metallic counterparts. Ti6Al4V (Ti64) alloy has emerged as a successful metallic biomaterial due to its characteristics including excellent mechanical strength and superior biocompatibility [4,5,6,7]. When compared with other metallic biomaterials, this alloy shows relatively low Young’s modulus, low density, high fatigue strength, and extraordinary corrosion resistance, which are the essential requirements for effective in vivo performance [8]. Porous Ti64 structures have been shown to be more successful in promoting cell growth as compared to implants with no porosity [9]. Porosity can be induced in two distinctive ways; the first approach incorporates the formation of internal porosity in the material by variation in powder size and the proper choice of process parameters [10,11,12], and the other approach uses geometrical cellular units that are repeated throughout the implant to create porosity [13,14,15,16]. The first approach, however, may lead to non-homogeneity of material distribution within the structure and cause catastrophic failure of the implants. In the second approach, control over porosity and tailoring of the properties are attainable in porous lattice structure (PLS) parts.

The complex PLS components are not possible to fabricate with conventional techniques. Additive manufacturing (AM) has the potential to fabricate such complex PLS and has the advantage of developing parts with two or more phases. These benefits make AM a favourable tool for the production of biomedical implants [17] even with the bone defect shape specific to each patient. ASTM grouped additive manufacturing into seven noteworthy processes including selective laser sintering (SLM) which directly produces end-utilize metallic parts with complex geometries [18,19]. In addition to this, in situ alloying and multi-metal processing are some of the benefits of SLM [20,21]. For intrinsic engineered features and complex geometry of the parts, SLM has been explored as a manufacturing technique in the biomedical domain. This technology has the advantage of manufacturing difficult PLS straightforwardly from a designed 3D CAD model with reduced production time [22]. Complex geometries such as the topology optimized structures and generative design structure are also possible to fabricate with SLM [23]. In the view of same, Erhard et al. [24] revealed that SLM technology produces parts with prevalent strength and lesser impurities when compared with conventional processes. Parts produced by the SLM technique, therefore, possess favourable mechanical properties due to the homogeneity of the material spread and the constant laser control [25]. Xuezhi et al. [22] examined the biocompatibility of SLM fabricated Ti64 porous plates through in vivo and in vitro tests and discovered favourable osseointegration. Literature review on SLM suggested that the greater part of the research revolves around the mechanical and tribological properties of the solid parts (with no porosity) [26,27,28].

Many researchers investigated the fabrication of complex geometries with AM [29,30]. These complex geometries have a unit cell structure of different geometries. Various unit cells including dodecahedron, tetrahedron, cubic, and gyroid, etc. can be used to induce porosity and make biocompatible implants [31]. Geometric dimensions of the porosity (i.e., strut length and pore size) influence the mechanical properties of the fabricated structure. PLS with pore size in the range of 100–1000 μm allow improved integration with host bone tissue, even bone distribution, and vascularisation [32]. Larger pore size improves permeability, which promotes bone ingrowth, but small pores are more suited for soft tissue ingrowth [33]. In terms of unit cell geometry, triangular, rectangular, and elliptic pores promote angiogenesis and induce faster cell migration due to their greater curvature, while staggered and offset pores help to create a larger bone volume as compared to scaffolds with aligned patterns [34]. Larger pore sizes are associated with higher compressive modulus [35]. The potential of gradient porosity scaffolds to preserve and restore their elastic properties after deformation is their key advantage, while square pores help to increase the stable mechanical strength [36]. 

Recent studies discussed the use of FE analysis for computing the mechanical strength of such PLS [37,38]. In a similar attempt by Mehboob et al. [39] FE analysis of porous Ti64 femoral stems with body-centered-cube (BCC) structure was conducted in ABAQUS. The results were compared and showed good agreement with experimental compression testing. Similarly, Mircheski et al. [40] developed and tested porous acetabular cups using 3D FE software. FE analysis was proved the best alternative to experimental analysis due to the early stage of the design and the development of porous implants for a relatively short time and reduced price.

Since the PLS usually performs under compressive loading, it is important to understand the relationship between its unit cell characteristics (pore size and strut thickness) and strength. As observed during the literature review, the cubic PLS components with different geometrical dimensions of the solid struts have not been explored for the mechanical properties. Thus, the work highlights the dependency of the maximum strength of the cubic PLS on the unit cell characteristics with tunable density for the target hard tissue replacement application. Specifically, an investigation was conducted to evaluate the effect of pore size and strut thickness on the mechanical properties (compression strength and elastic modulus), dimension accuracy, and fracture mechanisms using finite element analysis and experiments. This may help in achieving more mechanobiological properties required for human bone implants.

## 2. Materials and Methods

### 2.1. Materials

**Figure 1:**
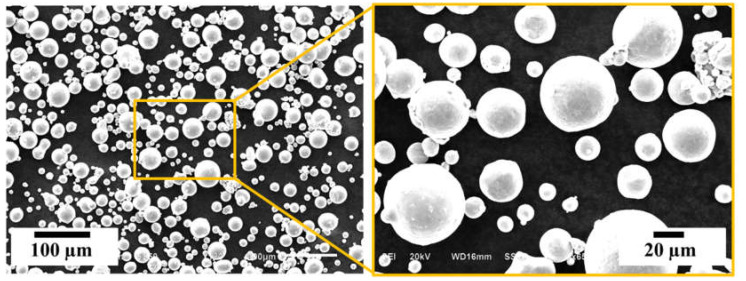
SEM images showing the morphology of Ti6Al4V gas atomised raw powder used for the fabrication of porous lattice structures.

Titanium alloy, Ti64 powder (EOS GmbH, Maisach, Germany) was used for the fabrication of the PLS components. The powder was produced using the gas atomization technique. In this process, the melted Ti64 ejects from a nozzle at the atomization bay and solidifies at a rate of 1000 K/s. Thereafter, the solidified powder gets cooled under vacuum followed by backfilled with inert gas. The composition of the Ti64 metallic powder having an average particle size range of 5–50 µm, used in this study, is given in Table 1a. This composition is as per ISO 5832-3 and ASTM F1472 standards. The powder morphology is shown in Figure 1.

### 2.2. FE Analysis

To ascertain the reasonable limits of unit cell parameters, i.e., pore size and strut thickness, PLS of different combinations of these parameters were designed using SolidWorks 2016 (Dassault System) package as shown in Table 2.

**Figure 2:**
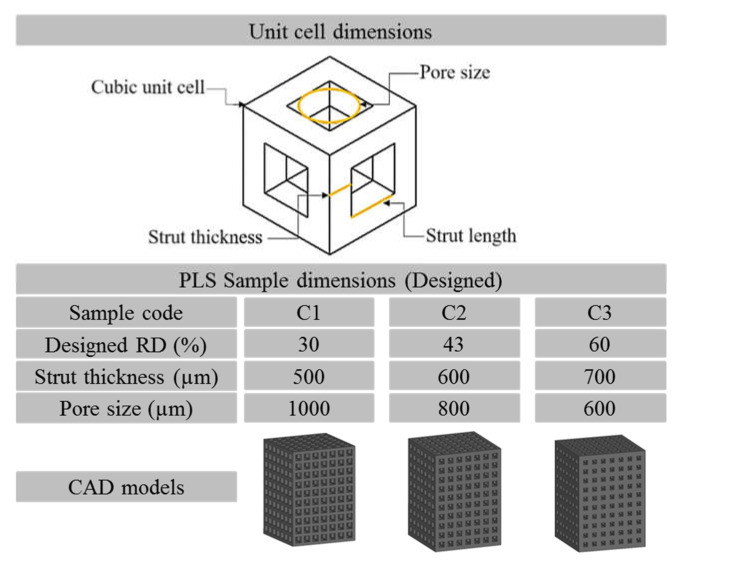
Specifications of the selected CAD designed samples.

Stress distribution and respective deformation during uniaxial compressive behaviour of PLS were analysed using ANSYS Workbench software. Rectangular samples of diameter 10 mm and height 15 mm (ISO 13314:2011) were designed using SolidWorks 2016 (Dassault System) package. The total number of unit cells in every PLS kept constant. In the FE analysis, the material properties used for Ti64 are shown in Table 1b. For the boundary conditions, the compressive pressure of 73 MPa was applied on the surface at top of a PLS and the bottom surface was constrained in all directions [41]. Solid standard mesh (that used Voronoi-Delaunay triangulation) with an element size of 1.24322 mm and a total number of 19,908 elements with a tolerance of 0.0621611 mm for each sample was used for the FE analysis [42]. The specifications of the computer on which FE analysis was performed are Processor-Intel(R) Core(TM) i5-6200U CPU@2.30GHz; RAM-8.00 GB (Notebook, Hewlett-Packard, California, USA). FE analysis was used to evaluate the stress concentration by inducing micro-porosity in the strut during compression of the single unit cell. FE analysis leads to the selection of three types of cubic PLS (i.e., C1, C2, C3) as shown in Figure 2. Three samples of each type, i.e., a total of 9 samples were fabricated using the SLM technique. PLS with an average pore size of 1000, 800, and 600 µm were considered as a result of the FE analysis in the present study.

### 2.3. Fabrication of PLS 

**Figure 3:**
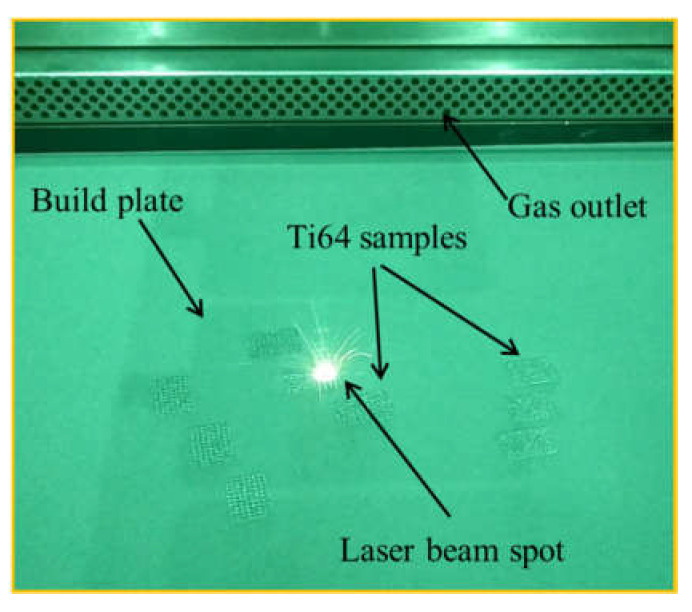
Fabrication of Ti6Al4V porous lattice structures by EOSINT M280.

In this study, the DMLS machine (EOSINT M280, EOS GmbH, Maisach, Germany) was used for the fabrication of PLS samples as per the schematic shown in Figure 3. To manufacture the samples, the first layer of Ti64 powder was spread on a preheated (100 °C) metallic build plate by a roller-based recoater mechanism. The thickness of the powder layer was kept at 30 µm. Selective portions of the powder layer were then melted by a focused laser beam that scans across the surface based on the defined model. A fibre laser with a wavelength range of 1.06–1.08 µm was used. To avoid oxidation of metal during the melting, the whole process was carried out in an argon atmosphere. Details of process parameters and other specifications are summarized in Table 3.

### 2.4. Evaluation of Morphology, Porosity, and Mechanical Properties

Pore size and strut thickness were measured using Tool makers microscope (Radical Scientific Instruments, Ambala, India; Model: RTM-99). Pore morphology, structural integrity, and internal defects were investigated using a scanning electron microscope or SEM (Model: 6610LV, JEOL, Tokyo, Japan). The relative density of PLS was determined by Archimedes’ principle using a weighing instrument. The density of each PLS was calculated by dividing its mass in the air by the total volume of the bulk structure. Then, the obtained density of PLS was divided by 4.43 g/cm^3^ (theoretical density of bulk Ti64) to obtain the value of relative density. Compression testing was accompanied by a mechanical testing machine (5985 200 kN load cell, Instron, Norwood, MA, USA) with a strain rate of 0.05 mm/min. The tests were performed in accordance with the ISO 13314:2011-standard for the mechanical testing of metallic porous materials [43]. The stress–strain curves were generated for each testing sample by taking an average of three values. The elastic moduli were also calculated. The testing was performed and data for force and displacement were collected. As the force is further increased, the sample snapped with the fixture emitted a cracking noise. The test was stopped at this point. Furthermore, the fracture mechanisms were analyzed by SEM images taken from the fractured samples, after compression tests.

## 3. Results and Discussions

The results of the investigations are presented in the order: first, results of FE analysis are presented which helped in the selection of suitable geometries of PLS that withstands the maximum load during its application, then the morphological characterization of the fabricated PLS was discussed to evaluate its replicability towards its designed models. Finally, the mechanical properties of the PLS were evaluated followed by the evaluation of fracture mechanisms of the samples that failed during compression testing.

### 3.1. FE Analysis

**Figure 4:**
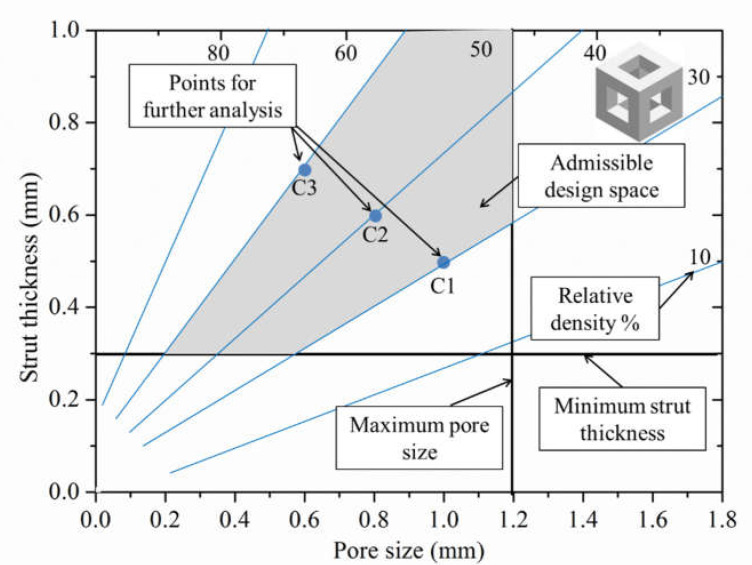
Result of FE analysis defining the admissible design space and points of further analyses constrained by strut thickness, pore size, and porosity percentage.

FE analysis helped in deciding the limits of lattice structure parameters which lead to the favourable mechanical properties compatible with the human bone. The rationale was made based on von Mises stress distribution and total deformation of the PLS components. The results of the study suggested that the pore size > 1.2 mm and the strut thickness < 0.2 mm lead to the generation of PLS samples having porosity percentages >60% which in turn have very low strengths (<20 MPa). These low values are not acceptable for hard tissue replacement alternatives as the strength under compression of the different human bones varies between 50 and 210 MPa [44,45]. The results of the FE analyses are shown in Figure 4. Based on the results obtained, only three types of PLS, i.e., C1, C2, and C3 were further investigated.

**Figure 5:**
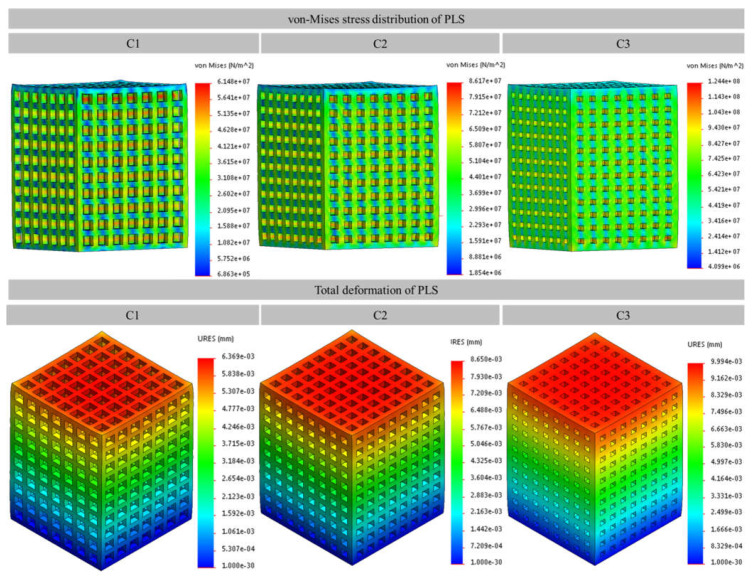
von Mises stress distribution and the total deformation of porous lattice structures evaluated using FE analysis (ae-b is scientific notation to a × 10^−b^); C1, C2, and C3 are the samples as per Figure 2.

The von-Mises stress distribution (Figure 5) showed that stress was primarily concentrated in the centre of the vertical struts in all the PLS samples. However, the intensity of stress decreased in the order C1 > C2 > C3. This is mainly due to the differences in design parameters of the PLS samples. It can be perceived in Figure 5 that the C3 structure exhibited maximum von Mises stress (107 MPa) as compared to C1 (61 MPa) and C2 (86 MPa). Besides, the deformations of 0.006369 mm, 0.00865 mm, and 0.00994 mm were observed for C1, C2, and C3 structures, respectively (Figure 5). The deformation was concentrated mainly at the upper layers of the cubes for all three structures.

**Figure 6:**
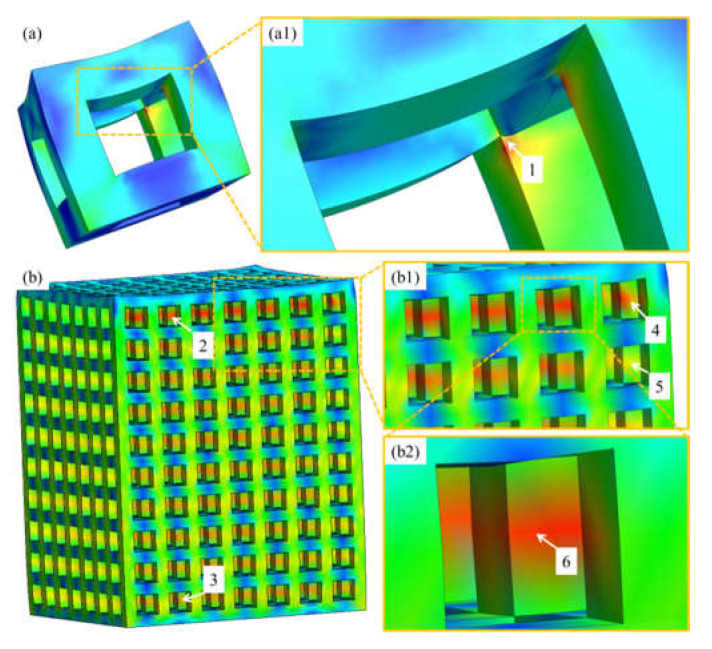
von-Mises stress distribution showing single unit cell (**a**), magnified view (**a1**), porous lattice structure (**b**), magnified view (**b1**), site of stress concentration (**b2**).

The internal corner points of a single unit cell were shown to be the weakest and point of fracture (Point 1 in Figure 6a,a1). However, when unit cells were replicated to form a PLS, the internal corner points got strengthened by the adjacent cells and provided resistance to failure under loading conditions (Point 2 in Figure 6b). In such a case, the vertical struts were the least supported, and hence the probability of failure at the middle point was maximum (Point 6 in Figure 6b2). In addition to this, it can be observed that the struts present at the outer boundary (Point 4, 5 in Figure 6b1) and at the lower portion of the structures (Point 3 in Figure 6b) were less prone to failure due to the development of less strain at those points. Pore size and strut thickness influence the mechanical and biological properties of the PLS components [46]. The relation between these parameters and the mechanical properties of PLS has been studied previously [47]. Our study also demonstrated different stress distributions of the PLS samples with different pore characteristics. Additionally, the von Mises stresses sustained by the PLS component increased with the increase of strut thickness, which is desired for an implant to withstand loading conditions. Furthermore, the pore parameters can affect biological performance. For example, with a large pore size >1000 µm, the RD of the PLS decreases which reduces the bone growth and adhesion [31]. On the other hand, with an increased strut thickness, the surface area increases, which enhances bone growth. 

### 3.2. Morphological Characterization

**Figure 7:**
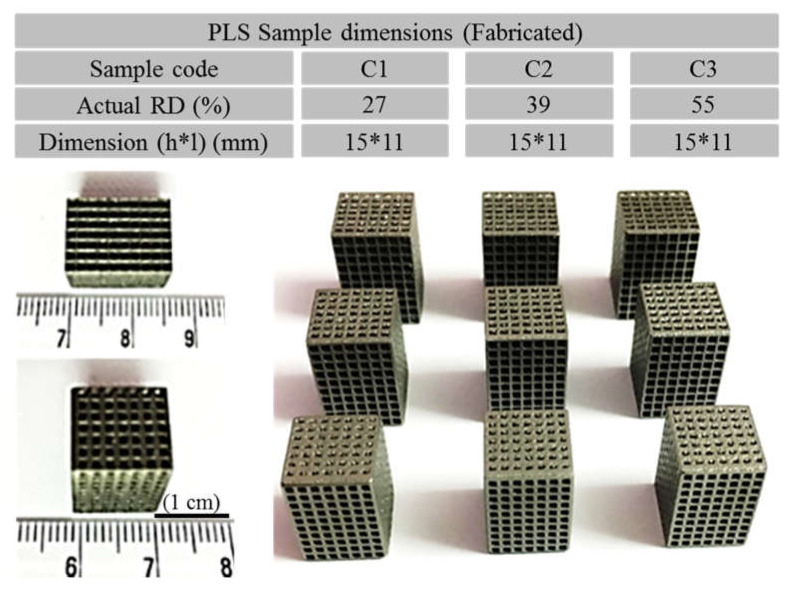
Dimensions of Fabricated cubic porous lattice structures by selective laser melting technique.

**Figure 8:**
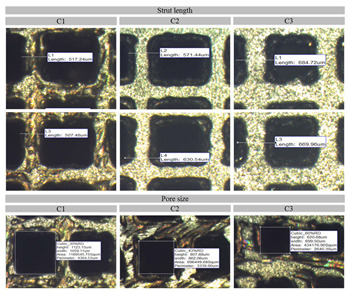
Optical images of fabricated porous lattice structures and their measured actual dimensions.

**Figure 9:**
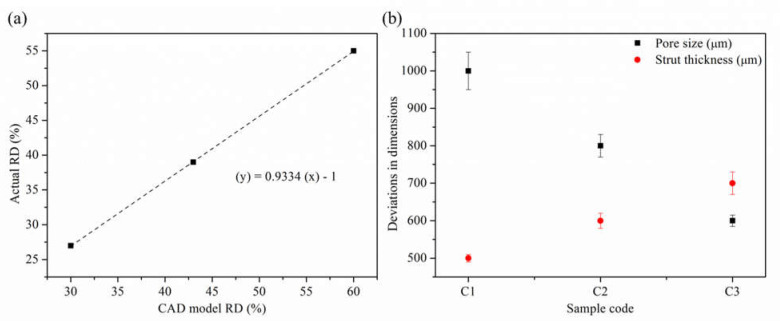
Relationship between actual and designed relative density (**a**), deviation in actual dimensions (µm) from the designed ones (**b**).

Figure 7 shows the successfully fabricated cubic PLS samples with different unit cell dimensions using the SLM technique. It can be observed from the fabricated PLS parts that they appropriately mimicked the designed structures. Furthermore, the optical microscopic images (Figure 8) confirmed the successful approximation of the fabricated structures by comparing the dimensions with those defined in the CAD model. The fabricated PLS samples had a dimensional variation of both strut thickness and pore size in the range of 2–6% (Figure 9b). This variation is primarily due to the shrinkage that occurred in the solidified part. Meanwhile, variation can be due to the system capabilities, i.e., the minimum spot size of the laser beam [48], part geometry-stair case effect [49], and powder characteristics [50].

It was found that the actual pore size was increased and the strut size was decreased in comparison to the designed structures (Figure 9b). During the inspection of PLS samples, it was observed that the struts had micro-pores mainly at the lower sections of the PLS components. This can be due to the presence of unsintered particles and hence improper melting [51]. Moreover, the temperature of the base plate can be a reason for the higher porosity at the lower sections. Relative density (RD) of the PLS samples was measured and it was in the range of 27–56%. It is important to mention that the actual value of RD showed a decrease of an average of 7–8% as compared to the designed PLS and followed a linear relationship as shown in Figure 9a. This variation can be attributed to the increased pore size and decreased strut size. This in turn is primarily due to the shrinkage and system capabilities as discussed above.

### 3.3. Mechanical Characterization

**Figure 10:**
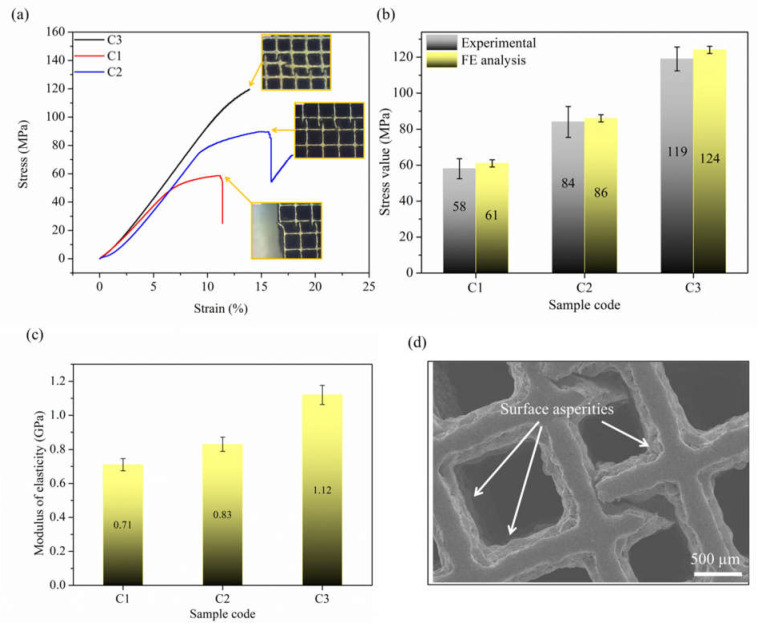
Stress–strain curves of porous lattice structures and their deformation behaviour during compression testing (**a**), compression strengths (**b**), modulus of elasticity (**c**), and a failed structures (**d**).

The compression stress–strain curves of the fabricated PLS samples are shown in Figure 10. Each stress–strain curve behaved like typical metal foam, similar to previously reported data [52]. It can be observed that larger strut thickness resulted in a larger collapse load. The stress was found to be increased linearly up to a certain value. After this value, i.e., the limit of proportionality, its slope decreased. At this point, the strut started bending. Further increase in load caused the strut to break with a snap noise. The number of unit cells in every PLS was equal. This was maintained by increasing the strut size and simultaneously decreasing the pore size during the designing stage. However, as the pore shape was the same, the difference in the compression properties can be attributed to the strut size. Thus, as it can be seen from Figure 10a when the strut size increased, the UCS increased as well. This increment was due to the rise in RD as strut size was increased. Furthermore, the experimental results validated the FE analysis, as shown in Figure 10b. It can be seen that the experimental values of UCS were 4–5% less than the predicted FE values. This error is likely due to the elimination of micro-pores and other surface asperities in FE design samples which were observed in the actual samples (Figure 10d). In addition to this, the elastic modulus of C3 PLS samples is in the range of about 1.12 GPa, which covers the range of scaffolds for human bone repair applications [53].

### 3.4. Fracture Mechanisms

**Figure 11:**
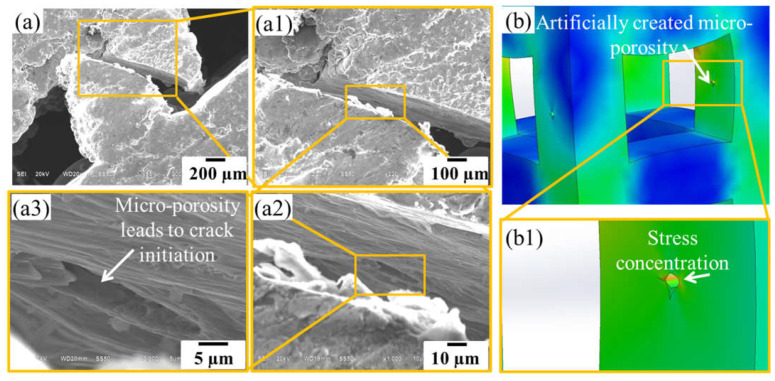
Crack initiation site inside the strut during compression loading (**a**, **a1**, **a2** and **a3**), stress concentration at the site of artificially created micro-porosity having the size of 50 µm (**b** and **b1**).

**Figure 12:**
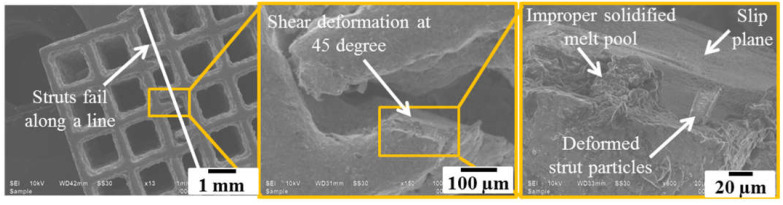
Failure of the vertical strut along a plane at 45 degrees.

As discussed in the previous section that the fabricated struts contained micro-level porosities in lower sections which were the crack initiation point, as revealed by SEM analysis shown in Figure 11. The SEM images of the failed struts during the compression test are demonstrated in Figure 12. It can be observed that when a strut failed, all the neighbouring struts existing in the same plane also failed. The mechanism of failure is usually dependent upon the cell geometry [54]. For instance, in bending-dominated PLS components made of diamond and dodecahedron unit cells, failure occurs along shear bands at 45 degrees to the PLS as the struts are at an angle to the loading direction hence experience shear failure [55].

However, in stretch-dominated PLS components like those made of cubic unit cells, layer-by-layer failure occurs due to the buckling as the direction of struts is parallel to the loading direction. Hence, stretch-dominated structures are ought to have superior mechanical characteristics as compared to bending-dominated structures [56]. As in the present case, the cubic unit cell failed along the vertical strut, which carried the maximum load during compression loading. From Figure 12, it is evident that the strut failed along a plane at 45° but the whole structure failed layer-by-layer along a line. Additionally, the brittle fracture of the struts was observed. However, the struts with larger sizes exhibited more plastic deformation. It was seen that the struts failed from the centre, this is due to the unavailability of the support structures as observed at the corners of the cubic unit cells. This automatically leads to the failure of the strut from the centre as it acts as the weakest point.

A comparative study was performed to associate the properties of the fabricated PLS samples here, with the existing literature. Table 4 shows the comparison of PLS mechanical properties based on various types of unit cells. It can be perceived from Table 4 that cubic PLS (C3) possesses mechanical properties close to that of other PLS components proposed for hard tissue replacement alternatives [53,55,57,58,59]. However, the mechanical properties vary at different porosity ranges. The cell proliferation data suggested that tetrahedron [59], dodecahedron [58], and cubic [60] with central spherical cavity geometry are far better than others [61].

Thus, it may not be appropriate to conclude the best lattice structure based on the mechanical properties only. The biological testing, however, is not in the scope of the present study, thus, the combination of pore size (600 µm) and strut thickness (700 µm) for cubic PLS is considered as mechanically feasible geometry irrespective of the variation in actual and designed dimensions. However, these variations can be eliminated by keeping compensation factors (fillets on sharp edges & corners and increment of strut thickness by 2% to avoid the shrinkage and improper melting in actual samples) during the designing stage as suggested by Bartolomeu et al. [35].

## 4. Conclusions

The different cubic PLS samples fabricated via the SLM technique were evaluated for dimension accuracy, compressive strength, elastic modulus, and fracture mechanisms. The main conclusions drawn from the present investigation are summarized below:

- FE analysis was used to define the design limits by measuring von Mises stress distribution and total deformation leading to the selection of suitable unit cell parameters for PLS manufacture.
- Cubic PLS samples with a relative density of 27–56% were successfully fabricated via the SLM technique. This could be attained by varying unit cell dimensions keeping the same number of unit cells in each PLS.
- In total, 7–8% variation was found in relative density of the fabricated PLS when compared with the designed model. This variation was linear and primarily due to the shrinkage and system capabilities.
- The maximum ultimate compressive strength was found to be 119 MPa of PLS with a pore size of 600 μm and an overall RD of 55%, which is comparable with that of human bone.
- The failure of the structure initiated from the micro-porosity formed during the fabrication process due to the improper melting. Furthermore, failure occurs in vertical strut along a plane inclined at 45 degrees.
- Making a decision on the best design parameters for bio scaffolds requires comprehensive research that contains both mechanical and biological aspects under the same conditions. Thus, the future study will concern the biological response of the proposed PLS. This will make the clinical use of these PLS more reliable and safe.

## Figures and Tables

**Table 1:** Elemental composition of EOS Ti6Al4V alloy powder (**a**), material properties used for FE analysis (**b**).

(a)								
**Element**	**Ti**	**Al**	**V**	**Fe**	**O**	**N**	**C**	**H**
**Contribution (wt.%)**	Balance	5.5–6.5	3.5–4.5	≤0.30	≤0.20	≤0.05	≤0.08	≤0.015
**(b)**								
**Property**				**Value**			**Unit**	
Elastic modulus				104.8			GPa	
Poisson’s ratio				0.31			-	
Tensile strength				1050			MPa	
Yield strength				827.37			MPa	
Mass density				4.4			g/cm^3^	

**Table 2:** CAD designed geometries of Ti6Al4V cubic porous lattice structures to be tested by FE analysis.

S.No.	Pore Size (µm)	Strut Thickness (µm)	RD (%)	Unit Cell Size (µm)
1	200	500	70	1200
2	400	400	48	1200
3	600	300	29	1200
4	800	200	10	1200
5	400	800	77	2000
6	600	700	60	2000
7	800	600	43	2000
8	1000	500	30	2000
9	1200	400	20	2000
10	1400	300	8	2000
11	900	1000	55	2800
12	1200	800	43	2800
13	1400	700	35	2800
14	1800	500	12	2800

**Table 3:** Process parameters used for the fabrication of Ti6Al4V porous lattice structures via EOSINT M280.

Parameter	Value	Unit
Scan speed	1200	mm/s
Hatch spacing	0.14	mm
Layer thickness	0.03	mm
Laser power	280	W
Laser diameter	80	μm

**Table 4:** Comparison of mechanical properties of porous lattice structures based on different unit cells.

Unit Cell Geometry	Pore Size (µm)	Strut Thickness (µm)	Porosity %	UCS (MPa)	E (GPa)	Reference
Cubic	600	700	45	119	1.1 ± 0.2	Present study
Diamond	-	246 ± 17.9	-	115 ± 3	-	[57]
Diamond	600	-	66 ± 0.3	113	3.694	[55]
Dodecahedron	560 ± 186	-	66.4 ± 0.3	117.2 ± 1.1	3.49 ± 0.02	[58]
Octet-truss	1000	400	77	117.3 ± 5.9	2.5 ± 0.2	[53]
Tetrahedron	1000	400	84	100.65 ± 2.9	1.31 ± 0.0	[53]
Tetrahedron	480	240	70	120 ± 4	2.9 ± 0.1	[59]
Honeycomb	400	-	77.5 ± 0.43	107.1	-	[62]
Irregular pores	-	368 ± 81	69.5	122	3.3	[63]

## Data Availability

All data generated or analyzed during this study is included in this article.
